# Integrating evolutionary aspects into dual-use discussion: the cases of influenza virus and enterohemorrhagic *Escherichia coli*

**DOI:** 10.1093/emph/eoab034

**Published:** 2021-10-26

**Authors:** Noble Selasi Gati, Ozan Altan Altinok, Sriram Kumar, Verónica A Ferrando, Joachim Kurtz, Michael Quante, Stephan Ludwig, Alexander Mellmann

**Affiliations:** 1 Institute of Hygiene, University of Münster, Münster, Germany; 2 Department of Philosophy, University of Münster, Münster, Germany; 3 Institute of Virology, University of Münster, Münster, Germany; 4 Institute for Evolution and Biodiversity, University of Münster, Münster, Germany

**Keywords:** dual-use, enterohemorrhagic *Escherichia coli*, influenza virus, SARSCoV-2, evolution

## Abstract

Research in infection biology aims to understand the complex nature of host–pathogen interactions. While this knowledge facilitates strategies for preventing and treating diseases, it can also be intentionally misused to cause harm. Such dual-use risk is potentially high for highly pathogenic microbes such as Risk Group-3 (RG3) bacteria and RG4 viruses, which could be used in bioterrorism attacks. However, other pathogens such as influenza virus (IV) and enterohemorrhagic *Escherichia coli* (EHEC), usually classified as RG2 pathogens, also demonstrate high dual-use risk. As the currently approved therapeutics against these pathogens are not satisfactorily effective, previous outbreaks of these pathogens caused enormous public fear, media attention and economic burden. In this interdisciplinary review, we summarize the current perspectives of dual-use research on IV and EHEC, and further highlight the dual-use risk associated with evolutionary experiments with these infectious pathogens. We support the need to carry out experiments pertaining to pathogen evolution, including to gain predictive insights on their evolutionary trajectories, which cannot be otherwise achieved with stand-alone theoretical models and epidemiological data. However, we also advocate for increased awareness and assessment strategies to better quantify the risks-versus-benefits associated with such evolutionary experiments. In addition to building public trust in dual-use research, we propose that these approaches can be extended to other pathogens currently classified as low risk, but bearing high dual-use potential, given the particular pressing nature of their rapid evolutionary potential.

## Lay Summary

Scientific research is usually conducted for the good of humanity, e.g. to promote health and cure and prevent diseases. However, it can also intentionally be misused, and this potential is called ‘dual-use’. We discuss this issue with special emphasis on evolution exemplified with two pathogens in an interdisciplinary manner from a biological and philosophical perspective.

## INTRODUCTION

Research in microbiology and infectious diseases has contributed enormously to the improvement of living conditions of humans. On the other hand, however, findings in pathogen research run the risk of being misused to harm humans, the environment or the society at large. This ‘dual-use’ dilemma depicts the ‘double applicability’ of scientific findings for good or for harm [1, 2]. It includes any technological development or research that can be misused to cause harm. With regard to the life sciences, Dual-Use of Research of Concern (DURC) denotes research that is intended for benefit, but which might easily be misapplied to cause harm (WHO: https://www.who.int/publications/i/item/who-consultative-meeting-on-a-global-guidance-framework-to-harness-the-responsible-use-of-life-sciences (28 October 2021, date last accessed).

The risk of dual-use of scientific findings is particularly high for research on pathogenic microorganisms, for example, with respect to their transmissibility and virulence, and became a public reality with the anthrax attack in the USA in 2001 [3]. This assault raised questions about potential population-level harm to human beings, which had previously not been considered by ethics committees or institutional review boards. Later, the controversy over two experiments that used genetic engineering to make highly pathogenic bird flu more contagious in ferrets, a model organism for virus transmission in humans, brought the debate to a new level of awareness [4, 5]. Critics claimed the risk of a pandemic, if these highly pathogenic pathogens fell into the wrong hands, that is, intentional misuse, or got out of the laboratory unintentionally. The validity of these concerns became obvious in 2014, when four safety breaches in the US Centers for Disease Control and Prevention (CDC) and National Institutes of Health (NIH) laboratories led to a potential exposure of several persons to four different pathogens that cause anthrax, smallpox, avian influenza and Ebola [6, 7]. Dual-use research also entails scientific research to increase pathogenicity and resistance of pathogens against antimicrobial substances, or to create strains capable of circumventing diagnosis [8]. Moreover, advances in the genetic manipulation of pathogens have outrun many legal and ethical frameworks. Therefore, dual-use research of concern presents manifold problems in research ethics and public health policy.

Within the current system of classification, pathogens are divided into risk groups (RGs) 1, 2, 3 and 4, with RG1 posing the lowest and RG4 the highest risk, respectively, based on their virulence, public health threat and treatment availability. A risk assessment for handling agents belonging to these groups corresponds to biosafety levels (BSLs) 1, 2, 3 and 4, which include technical, organizational and personal protective measures. Pathogenic bacteria of RG3, e.g., *Bacillus anthracis*, *Yersinia pestis* and RG4 viruses, for example, Ebola and Marburg viruses, causing hemorrhagic fever, are regarded to be possibly misused in bioterrorism attacks [9–12]. While this assessment is correct, some RG2 pathogens are often on the evolutionary edge of becoming RG3, such as enterohemorrhagic *Escherichia coli* (EHEC; which are classified as RG3** in Germany [13]) and Influenza virus (IV), also demonstrate significant dual-use potential. These two pathogens should be of great concern as they have a high potential to spread in the human population and there are currently no effective treatment options. Moreover, recent outbreaks resulted in enormous media attention, leading to public fear and causing huge financial losses to business and healthcare institutions [14, 15].

In this article, we discuss matters related to the potential dual-use of IV and EHEC with special emphasis on evolutionary aspects, which seem to have been neglected in previous debates [16]. Pathogens with an intrinsic, natural ability to evolve fast may raise novel ethical concerns beyond the usually considered gain-of-function (GOF) experiments. We further explore the possible imminent biosecurity risk and discuss the responsibility and roles of researchers, from both scientific and philosophical perspectives, in assessing and reducing the risk of potential misuse and intentional release of these pathogens into the human population. This way, we aim to address the new challenges for research involving pathogens, which we denote in this context ‘Rapidly Naturally Evolving Pathogens’ (RNEPs), such as IV and EHEC.

## CURRENT ASPECTS OF THE DUAL-USE DISCUSSION

### Dual-use risk with directed engineering

*Case of IV.* Surprisingly, dual-use aspects were not an issue in IV research until quite recently. In retrospect, a few key research findings and events in influenza epidemiology and evolution can be identified that collectively caused enhanced dual-use awareness.

The 1918 H1N1 Influenza pandemic, commonly referred to as the ‘Spanish Flu’, caused around 0.5–1.0 billion infections and up to 100 million deaths during four waves of infection worldwide [17]. A characteristic feature of this virus strain was the high mortality it caused in healthy adults aged between 15 and 34 years. The pandemic lowered the average life expectancy in the USA by >12 years. Until today, comparable morbidity and mortality rates were not observed during any of the other influenza pandemics or seasonal epidemics. The high pathogenicity of this virus strain puzzled researchers for several decades, prompting questions such as ‘Why was this flu strain highly pathogenic?’, ‘Where did the virus strain originate from?’, ‘How can this virus strain potentially evolve?’ and ‘What can the public health officials learn from the 1918 pandemic to be better-prepared against future pandemics?’. Answering these questions required an improved understanding of the virus components, its evolutionary dynamics, and its infection epidemiology. After several unsuccessful attempts by different laboratories around the world, Neumann et al. [18] succeeded in completely assembling a replication-competent IV from cDNA in 1999, which was further developed and later employed to seek answers to the aforementioned questions [19]. An expert group of researchers ‘revived’ the virus strain from formalin-fixed lung samples of 1918 victims, sequenced its genome, recreated the whole virus in highly contained BSL-3 laboratories at the CDC, ultimately characterizing its biological features [20, 21]. While several research findings have shed some light on the peculiar features of the 1918 Spanish flu strain, the actual reasons for the high pathogenicity of this virus strain remain elusive [22]. Surprisingly, the intentional ‘revival’ of this virulent strain did not raise strong public concerns about its dual-use potential, partly due to the sparse awareness about the dual-use concept back then.

In 1997, there was an unprecedented outbreak of highly pathogenic avian H5N1 IV in Hong Kong, followed by its successive reemergence in 2003, which spread to multiple Asian and African countries (Box. 1). Although there was no recorded evidence of sustained human-to-human transmission back then, the high fatality rates associated with unusual symptoms of severe systemic inflammation raised serious public health concerns about the possible successful adaptation of the virus to humans and—as a consequence—its improved ability to spread among humans, possibly leading to a pandemic in humans. Evaluating the likelihood of these events required characterizing the biological relevance of the novel mutations found in these flu strains. This was experimentally addressed by two groups, which was the take-off point for an intense dual-use debate in IV research. The controversy started at the European Scientific Working Group on Influenza (ESWI) meeting in Malta in September 2011. A group from the Netherlands showed the creation of an H5N1 variant that was contagious between ferrets (the preferred animal model mimicking transmission among humans), and which differed in only five amino acid positions from the wild-type strain [4, 5]. This novel combination of mutations, each of which were already known from infection in birds in nature, suggested that H5N1 IV could in principle evolve to a pathogen that is highly transmissible among mammals, and particularly in humans. In parallel, a US laboratory performed similar yet safer experiments, using a portion of the H5N1 virus in a genetic background that was susceptible to antivirals and vaccine-induced immune responses [4, 5].

**Box 1.**
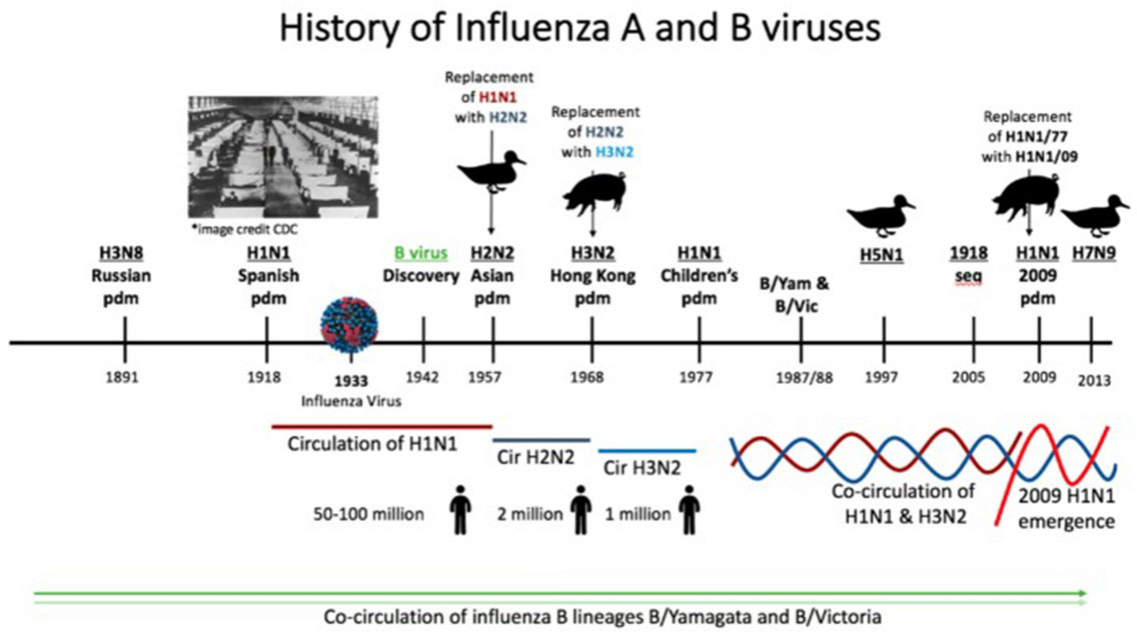
History of Influenza A and B viruses.

The awareness that had been raised by reports from the ESWI conference and the fact that the related funding of both laboratories was largely provided by the US government, brought the authorities into play. The two paper drafts were sent to the National Science Advisory Board for Biosecurity (NSABB). The board recommended publication of the work, however, with restricted access to sensitive portions of the manuscripts, available only to expert researchers in need of such information for further studies [30]. In parallel to the NSABB examination, 39 influenza researchers voluntarily agreed to a 60-day moratorium on research regarding H5N1 transmissibility, which was later further extended [31]. These incidences led to heated controversies in the media and among experts in the field [32], which later diminished due to novel findings [33]. However, the discussion of the dual-use dilemma persisted and led to a more general perception of the benefits versus the risks of research on dangerous infectious pathogens.

*Case of EHEC.* One major concern about the pathogenesis of EHEC is its ability to progress to hemolytic uremic syndrome (HUS) (Box 2), which is primarily caused by Shiga toxin (Stx), the major EHEC virulence factor [15]. The rate of HUS development can be as high as 20% for some wild-type EHEC infections [38]. The reservoir of EHEC is mainly cattle, and outbreaks are often linked to the consumption of animal products (Box 2). Genetic manipulation can consequently lead to the creation of strains having the ability to survive in different environments, leading to multiple sources capable of causing an outbreak. The intentional release of such virulent or engineered strains of EHEC into the human population would have a serious impact on the global economy, healthcare systems, and public confidence. An estimated 251 million Euros excluding health care expenditure on patients were lost in the EHEC 2011 outbreak centered in Northern Germany [15].

**Box 2.**
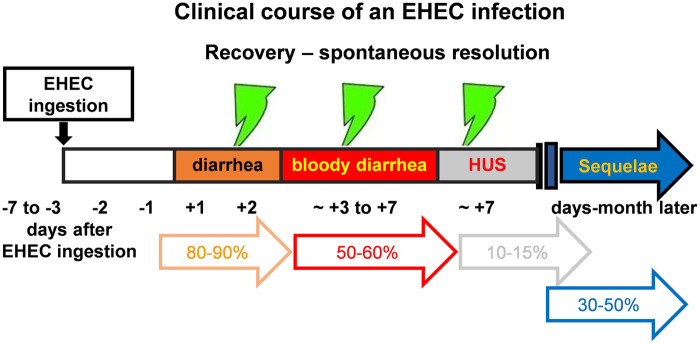
Clinical course of an EHEC infection.

A global outbreak due to the intentional release of an even more virulent EHEC strain could be more devastating, including approximately 30% of HUS patients suffering from long-term sequelae [45]. Companies could be harmed due to the boycott of their products linked to the outbreak. For example, during the 2011 EHEC outbreak, Spanish cucumbers were initially wrongly considered the source of the outbreak, leading to enormous losses to cucumber farming and a €2 million damage lawsuit filed by a Spanish vegetable company [46, 47]. Unfortunately, current knowledge on the pathogenesis of EHEC is still limited and further research is needed to better understand the epidemiology, pathogenesis and evolution of this pathogen. However, while scientific research is indispensable in containing and preventing an intentional release of this pathogen, the use of scientific information to create potentially deadly strains should not be overlooked. Furthermore, deadly EHEC strains generated with the available scientific knowledge could find their way out of the laboratory into the human population. Finally, the fact that even commensal *E. coli* are one of the most common lab-used bacteria (and are therefore fairly easily engineered), which can also naturally evolve toward highly pathogenic forms, genetic manipulations should also be critically evaluated since many organisms can be weaponized illustrating that dual-use is a broad concept to be considered.

Whereas research to enhance virulence of EHEC using directed engineering has, to our knowledge, not been conducted in the past, the scientific community was nevertheless very much interested in unraveling the relevant factors for increased virulence of certain EHEC clones, in particular of the EHEC O104: H4 clone causing a large outbreak in 2011. Here, factors that had led to the evolution of enhanced virulence in these bacteria were determined indirectly. Different studies could demonstrate that the presence of a single plasmid harboring fimbriae mediating the tight adhesion to intestinal epithelial cells was crucial for an efficient transport of toxins to the human host [48] and that—on the contrary—the *in vivo* loss of this plasmid decreased virulence [49]. Moreover, it was shown that exactly this type of fimbriae provided the most efficient adhesion to the host cells [50], illustrating that during the natural evolution of these strains it was very likely the occurrence of the worst combination of virulence determinants, that is, the Stx and the respective fimbrial subtype. Finally, even before the large outbreak in 2011, the gain and loss of relevant genetic material *in vitro* [51] and *in vivo* [52] were demonstrated, opening the door for potential misuse.

### Current regulatory frameworks

The need for ethical and legal frameworks to guide research activities led to the creation of the ethical committees seen today, which started with the Nuremberg code after the Second World War. The ethical concerns were later further deepened to address the growing impacts of research through the Declaration of Helsinki in 1964 [53]. Due to the legacy of these codes, for decades, debate on research ethics revolved mostly around research done on humans and other animals. Up to now, current legal and ethical frameworks are insufficient to handle recent research on pathogens by means of selective breeding and genetic engineering [54, 55]. Although there are classical concerns about freedom of science and regulation [56], the research output of some studies raises concerns due to the potential harm that can be caused using such scientific findings.

As the new potential for harm increased in relation to biological research, novel ethical and legal understandings were put forward [4, 5]. In the USA, the NSABB advisory committee was founded to address issues related to biosecurity and dual-use research [8]. Although NSABB recommends control over publication of scientific knowledge, due to strong objections from scientists, this position was rejected [55]. However, in 2013, a group of researchers working on vaccines petitioned the US president’s bioethics committee, defending DURC on IV and similar research that are ‘ethically and morally wrong’ [55, 57]. Furthermore, there have already been at least four safety breaches in labs as recently as in 2014 [6, 7]. With the increased number of research projects that can be categorized as DURC, the risk of intentional or unintentional security breaches raised to a new level. In addition to that, Potential Pandemic Pathogens research is becoming an even more pressing issue [53, 55]. Because of the increased risk and threat, changing the focus of research from GOF experiments was argued for [16]. Since the challenge is obvious, there have been discussions on how and to what extent there should be new regulations on research. Already due to the growing concern about the potential harmful use of some scientific findings, the ‘Fink report’, published by the National Research Council in 2004, called for voluntary self-governance of the scientific community in the life sciences field [8]. However, ‘it has been shown, for example, that life scientists generally lack awareness of the ways in which their well-intentioned research might be abused by malevolent actors and, indeed, that they lack awareness of the dual-use phenomenon in general’ [54]. Moreover, many scientists generally believe that scientific knowledge is ethically neutral or inherently good [8, 58].

## EVOLUTIONARY ASPECTS OF THE DUAL-USE DISCUSSION

### Dual-use risk of evolution research

The dual-use discussion on infection research currently revolves around GOF mutations and directed engineering of pathogenic microbes, while evidence suggests that the products (pathogenic variants) and outcomes (characteristic mutations) arising from experimental and clinical evolution, that is, RNEPs, are closely comparable. Therefore, the naturally/experimentally evolved pathogens may have equivalent pathogenicity and thereby comparable dual-use potential, and the experimentally evolved microbes might be generated through either mutational experiments or directed engineering. For example, H274Y substitution in the coding sequence of the Neuraminidase (NA) protein of IV confers resistance against the current first-line anti-influenza NA-inhibitor oseltamivir [59]. This substitution was observed in *in vitro* experimental evolution carried out by Hurt et al. [60] through successive passaging of the virus under oseltamivir selection pressure, similar to the natural evolution of IV in Vietnamese patients treated with oseltamivir [61]. This highlights that comparable pathogenic strains with characteristic mutations related to oseltamivir resistance result from both natural and experimental evolution. Another example is the combination of point mutations I222V and E119V in the coding sequence of the NA protein, making the influenza-A virus less susceptible to oseltamivir. These two point mutations were also described by Hurt et al. in their *in vitro* experimental evolution, in addition to the H274Y substitution [60], and it was also found after natural evolution of a H3N2 strain infecting an immune-compromised patient which received oseltamivir treatment [62]. Similar evidence was reported by Molla et al. [63] and Samson et al. [64]. Nguyen et al. comprehensively reviewed all such overlaps between the results of clinical, that is, intra-host *in vivo* evolution, and experimental evolution [65].

Similarly, the six amino-acid substitutions L26F, V27A, A30T (A30V), S31N, G34E, and L38F in the coding sequence of M2 (Matrix) protein of IV confer amantadine resistance and were selected during natural evolution of IV. Such mutations are now present in most of the currently circulating strains, even in the absence of amantadine selection pressure, making the current influenza treatment with amantadine ineffective [59]. Similar outcomes are expected for the NA-inhibitor oseltamivir, thereby requiring yearly surveillance to monitor seasonal IV strains which might carry oseltamivir-resistance mutations, even in the absence of oseltamivir selection pressure [42]. These examples highlight that results from both clinical and experimental evolution under comparable selection pressures bear the risk of the emergence of ‘unwanted’ variants that might facilitate dual-use harmful purposes.

### The imperative need for evolution research

In this area of conflict, we believe that the potential benefits and harm have to be carefully considered, particularly in the field of RNEPs. First, it is important to ask whether potentially harmful RNEPs could be replaced by less harmful bacteria or viruses for the experiments. If not, decisions may be based on the following questions: (i) Is the risk of the outcomes of natural evolution of these pathogens high enough to make us do research in the lab, for example, via experimental evolution? (ii) Can we predict the outcomes of natural evolution through *in vitro* experiments? And (iii) Is experimental research on evolutionary processes more important or at least more useful than results we can obtain from theoretical models, which could be based on either observational data (e.g. genome sequence and corresponding epidemiological data) or theoretical mathematical evolutionary models? The answers to these questions will facilitate the decision of whether the benefits of doing research on the evolutionary aspects of these pathogens will outweigh the risks of harm. In contrast to the questions (ii) and (iii), which warrant more extensive considerations in the forthcoming sections, the answer to the first question is easier in most cases. During the current severe acute respiratory syndrome coronavirus 2 (SARS-CoV-2) pandemic, for example, the answer to the first question is—at least for certain strains—definitely yes. For other microorganisms, which cause only little harm to humans or non-human animals, doing research, which can have potentially dangerous outcomes, the answer might be ‘no’ depending on the likelihood of a possible positive outcome of the research (no generation of highly pathogenic strains) versus the potential danger of such research to humans, animals or the environment.

When it comes to answering the second question, sometimes the usefulness of predicting new potential strains of pathogens is disputed. Evolutionary outcomes are often unpredictable [66] and the knowledge achieved with experimental evolution is thus not accomplishable through conventional genome engineering or GOF experiments alone [16, 67–69]. Moreover, research on pathogens is not only about their specific features, such as the presence of certain mutations. Rather, evolution experiments inform us about the mechanisms, that is, the evolutionary processes more generally [66], and thus possible evolutionary trajectories and even potential starting points for countermeasures, for the prevention of highly pathogenic strains. Moreover, in addition to general knowledge of evolutionary processes, understanding the working mechanisms in particular systems [48, 70, 71] and assessing likely evolutionary trajectories of a strain is crucial. Using as an example the EHEC O104: H4 from the HUS 2011 outbreak [38, 39], *in vitro* studies demonstrated that the presence of a specific plasmid that mediated tight adherence to intestinal epithelial cells was required for an efficient transfer of toxins into the human host [48]. In parallel, clinical observations corroborated this finding: EHEC O104: H4 strains that lost this plasmid during the 2011 outbreak were associated only with mild diarrhea [49]. Another example for EHEC is the ability of such strains to rapidly evolve by loss or acquisition of genetic material. Here, we learned from natural evolution that gene loss is a frequent phenomenon that can affect relevant toxin genes [49, 52, 72]. Furthermore, it was also demonstrated through *in vitro* experiments that toxin genes can be easily acquired under conditions that are likely to happen during *in vivo* natural or experimental infection [51]. A better understanding of the underlying mechanisms for such gain or loss of bacterial genes could help to develop novel approaches to prevent the progression from a mild to a severe disease, for example, by manipulating the rate of virulence gene loss during the early stage of an EHEC infection, to promote such loss and lower the likeliness of toxin gene acquisition.

This leads us to the third question of whether experimental research on evolutionary processes is more important or at least more informative than results we can obtain from theoretical evolutionary models that are built from experimental/observational/epidemiological data. As in most biological systems, host–pathogen interaction, coadaptation and coevolution are often multifactorial, and most theoretical models, although built on experimental/observational/epidemiological data, would have limitations in terms of not holistically capturing all/most influential factors, thereby leading to biased results. As an example of these limitations, during the ongoing SARS-CoV-2 pandemic, evolutionary trajectories of the different viral variants of interest and variants of concern could be explained through theoretical models built from ‘real world’ epidemiological data, with rather as-of-yet limited knowledge that could be derived from experimental results, given the biological novelty of the virus strain. However, attempts to predict which viral variants will be spreading in the future remain difficult, that is, experiments will be needed to complement theory based on epidemiological data [73]. Whereas the above-described biases primarily originate from missing experimental data, it has to be noted that even the availability of experimental data does not fully solve the dilemma, as most experimental approaches also have some limitations, for example the *in vitro* conditions can reduce the general applicability of results, and usually only a limited number of representative strains can be analyzed. This is, for example, the case, when circulating influenza-A and influenza-B strains are subjected to epidemiological surveillance to identify the most prevalent seasonal strains and to formulate accordingly the annual flu vaccine: while it is relatively easy to analyze the genome sequences of several circulating strains within an epidemiological model in order to narrow down to the consensus sequence of the most prevalent circulating strains, experimental approaches and epidemiological data are often required as complementary inputs to shed light on the evolutionary trajectories of the circulating strains [74]. On the other hand, although the surveillance-based/epidemiology-enabled theoretical models help explain the past trajectories, ‘pure’ experimental data is required to predict/understand the full future picture. For example, Shi et al. studied the selection pressure on hemagglutinin (HA) genes of H9N2 IV from different hosts, under controlled and ‘natural’ evolution scenarios [75]. Although they could detect some common features in IV after evolution in the different hosts and conditions investigated, they also found host-specific outcomes that were ‘process-centric’, that would unlikely have been predicted by only using theoretical models built from experimental outcomes, which would not have capitulated these process-driven factors. This underlines the importance of studying evolutionary processes experimentally, to avoid biased results due to the use of incomplete theoretical models. Based on these findings, preventive interventions to possible epidemics or pandemics may become feasible through anticipating the potential evolutionary pathways of these microorganisms. Such research, although in general regarded to be far from applicable, also harbors dual-use risk, since such scientific knowledge could also be used for harm [76].

There needs to be a fundamental acknowledgement that experiments involving *in vitro* or *in vivo* evolution under selection pressures that involve highly pathogenic microbes—either at the beginning or at the end of the experiment—harbor the risk of potential dual-use. The advocates of the scientific community’s self-control on the situation argue for an increased regulatory network but within the scientific community. As indicated in the American Medical Association’s 2005 ‘Guidelines to Prevent the Malevolent Use of Biomedical Research’, life scientists are expected to be responsible for the outcomes of their research [53, 77]. This analysis is a good starting point for encouraging scientific community-centered decision-making structures and to enforce rules of conduct and regulation of the scientific community via professional training of life-scientists similar to medical doctors [78, 79]. Establishing committees that include representatives of different groups, such as life scientists, public policy agents, biosecurity experts and civilians for assessing dual-use research is especially important and should always be mandatory [54]. But we claim that scientists should have a strong influence on this decision-making mechanism. Due to their expertise in understanding the possible outcomes of their own research, for example, involving IV and EHEC, researchers should not only take part but also especially take responsibility in the prevention of potential dual-use.

## CLOSING REMARKS

While we have encountered the dual-use issues in the field of IV and EHEC evolutionary research in the past, at the time of writing, the ongoing SARS-CoV-2 pandemic completely overwhelmed the scientific community and society more generally. One specific point with an evolutionary perspective was the public debate on the origin of the SARS-CoV-2 virus. The initial uncertainty supported conspiracy theories that the virus has been bioengineered and originated from a laboratory, which put the dual-use dilemma in the spotlight [80–83]. Fortunately, evolutionary studies could trace back the SARS-CoV-2 outbreak to an initial zoonotic event, originating from horseshoe bats through an unknown intermediate host in early November 2019, followed by its initial outbreak within the wildlife market of Wuhan, China [84, 85]. This example of the origins of SARS-CoV-2 shows that research into the evolution of a pathogen can be important to clarify the most likely source of a new variant causing infection in humans. From a societal standpoint, these scientific clarifications had enormous impact as they helped to refute misinformation and conspiracies that the virus emerged from a biosafety breach from a laboratory or—even worse—was intentionally released as part of a bioterrorism act. Interestingly, also during the large EHEC 2011 outbreak, the likelihood of a bioterrorism attack was discussed and further increased public fear. Again, in-depth epidemiological and evolutionary investigations clarified the source of the outbreak and helped to increase the public trust in science. This trust is necessary to prevent and especially to fight pandemics and epidemics. It is therefore imperative to discuss publicly and among scientists dual-use policy and ethics of evolutionary research on pathogens to increase public trust and to further counteract misinformation and conspiracies. The contribution from diverse actors in an interdisciplinary way, for example, by answering the suggested questions (i, ii and iii), is crucial to increase awareness and responsible behavior in science and research, to ultimately promote a free, well-informed society and the sustainability of democracy.

## GLOSSARY

**Potentially pandemic pathogens (PPP).** Pathogens that are highly transmissible and virulent, cause significant morbidity and/or mortality and are capable of wide uncontrollable spread in human populations.

**Rapidly naturally evolving pathogens (RNEP).** Pathogens have the intrinsic potential to evolve quickly under natural conditions even during infection of a single host. They may possess unstable genomic backbones and/or infidel replicating machineries that altogether make them highly susceptible to genomic changes and thus may exist as diverse species or quasi-species.

**Gain of function (GOF).** Gain-of-function research (GoF research or GoFR) is research that genetically alters an organism in a way that may enhance the biological functions of gene products. This may include an altered pathogenesis, transmissibility or host range, that is, the hosts that a microorganism can infect. This research is intended to reveal targets to better predict emerging infectious diseases and to develop vaccines and therapeutics.
